# An interpretative phenomenological analysis of the experience of couples’ recovery from the psychological symptoms of trauma following traumatic childbirth

**DOI:** 10.1186/s12884-022-05091-2

**Published:** 2022-10-29

**Authors:** Rosie Attard, Jane Iles, Florence Bristow, Rose-Marie Satherley

**Affiliations:** 1 School of Psychology, Department of Psychological Interventions, University of Surrey, GU2 7XH Guildford, UK; 2 South London & Maudsley NHS Foundation Trust, London, UK

**Keywords:** Postnatal mental health, Birth trauma, Interpretative phenomenological analysis

## Abstract

Globally, a large proportion of birthing mothers, and a to a lesser extent their partners, experience birth trauma each year, and yet access to adequate post-natal trauma support is rarely available. Untreated birth trauma has been shown to negatively impact the family in terms of the parents’ relationship with one another, and long-term negative consequences for the child. Despite a drive towards integrating mental health support into maternity services and a call to provide mental health support for couples rather than solely the birthing mother, there is little research exploring what birthing couples find helpful in recovery from birth trauma. The current research interviewed six couples using an Interpretative Phenomenological Approach in order to explore their understanding of what supported their recovery from birth trauma. Four themes were identified: ‘We need validation’, ‘Feeling paper thin’, ‘This is a system failure’ and ‘Birth trauma is always going to be a part of you’. The data describes an understanding of parents’ feelings of vulnerability and loss of trust in services to provide support following birth trauma. Further, parents’ need for validation and repositioning of control away from healthcare professionals when considering the availability and knowledge of the support options available is discussed. Clinical implications for supporting parents following birth trauma are explored, including an identified need for trauma informed care communication training for all healthcare professionals involved in maternity care, and the requirement for sources of therapeutic support external from the parent dyad in order to maintain the couples’ interpersonal relationship.

**Supplementary Information:**

The online version contains supplementary material available at 10.1186/s12884-022-05091-2.

## Background

Trauma, as defined by DSM-V [1] is comprised of perceived threat, and emotional response. Approximately 14.3% of mothers experience these two criteria after experiencing a traumatic childbirth, however when purely considering the threat aspect, it is thought that almost 30% of mothers meet this criteria [2]. Factors associated with traumatic birth include obstetric complications, first time births, emergency caesarean section, as well as fear, lack of control, negative appraisal of past deliveries, negative ratings of healthcare professionals, and interpersonal difficulties [2–4]. Leinweber et al. (2022) developed a woman centered definition of birth trauma, “A traumatic childbirth experience refers to a woman’s experience of interactions and/or events directly related to childbirth that caused overwhelming distressing emotions and reactions; leading to short and/ or long-term negative impacts on a woman’s health and wellbeing.” This definition highlights the importance of healthcare provider interaction in the experience of trauma for birthing women [5].

Access to trauma support, which can include both psychological therapy and psychotropic medication, is associated with better outcomes for the mother and the child. Specifically, reduction of psychological distress, the child’s social-emotional development [6], the mother-baby bond, and the mother’s satisfaction with their spousal relationship [7]. Research and clinical intervention have predominantly focused on trauma support for mothers, with little support for the birthing partner. This is problematic, as traumatic birthing experiences can also influence the birthing partner [8]. When one person within a couple experiences symptoms of PTSD following a traumatic birth, the other partner is more likely to also experience symptoms [9]. Up to 7% of fathers and partners are thought to experience symptoms of PTSD following bearing witness to a traumatic birth [10]. Critically, the couple’s interpersonal relationship is an important area of consideration when understanding recovery from PTSD after a traumatic childbirth. Little is known about how couples recover following traumatic childbirth, and what support may be helpful in overcoming psychological symptoms of trauma within a dyad during this period. However, theories have arisen citing communication between the couple as vital for recovery from PTSD, allowing processing of the trauma [11]. For example, Sautter et al. [12] found that Iraq veterans and their partners found joint recovery from trauma symptoms using ‘empathetic communication skills’, reporting the importance of couples’ connectedness and communication. Furthermore, an adaptation of the ‘systemic traumatic stress model’ for couples has been developed [13], highlighting ‘chronic stress, attachment, identification, empathy, conflict and physiological responses’ as the mechanisms of systemic trauma within a couple.

Internationally, there are calls for mental health care to be improved during the perinatal period, and for support to be integrated into maternity services [14]. International studies of the status quo of support available following traumatic childbirth demonstrate that only one country, the Netherlands, holds national policies on the ‘screening, treatment and prevention of a traumatic birth experience’. Most of the 18 countries included provided a form of ‘informal support’ however this was not substantiated with formal training for those delivering the support [15]. Despite the movement towards perinatal mental health provision to extend to couples rather than purely the birthing mother, there is very little evidence to suggest what helps couples – rather than just the mother - recover from psychological symptoms after traumatic childbirth. The literature that focuses on recovery has identified a gap in qualitative approaches to identify what couples’ themselves find helpful [16]. Therefore, this study explores couple’s experiences of trauma following childbirth, and their journey to recovery. Specifically, the focus is on how the couple interact with each other and the health system during this time.

## Methods

This study uses Interpretative Phenomenological Analysis (IPA), an approach that seeks to generate contextualized knowledge, to generate novel insight into participant experiences [17]. The data presented here were captured from in-depth, online interviews with couples.

### Participants

Couples who self-reported that at least one member of the dyad found their labor and/ or childbirth experience traumatic, were invited to take part. The childbirth was required to be within 6 months to 2 years prior to the interview; this timeframe was to allow for processing of the trauma to enable discussion around potential recovery, however within recent memory [10]. This time-period also aligns with the extension of perinatal provision in the UK, to support families up to two years postnatally. A full list of inclusion and exclusion criteria can be found in Table 1.

**Table 1:** ; Inclusion and Exclusion Criteria

Inclusion criteria • Couple experienced a traumatic childbirth, as determined by the mother/partner. • Childbirth occurred between 6 months and 2 years before participation. This was included to allow for processing of the trauma enough to be able to talk about potential recovery support (as in Ayers, 2007), however not so long ago that couples can’t remember the experience. This also fits in with the new model of perinatal services working, as individuals may soon still be under the care of the team up until 2 years after the birth of their child. • Both members of the couple able to provide informed consent • Both members of the couple over the age of 18 years old • Both members of the couple willing to consent to take part	Exclusion criteria • Baby has died • Baby has spent time in neonatal intensive care or special care; this is due to the nature of this being traumatic in itself, and making the focus on recovery from birth trauma less clear in the research. • No known child protection cases ongoing for the parents • One/both of the parent’s fluency of English does not allow for an interview • One/both of the couple lacking capacity to provide informed consent

### Procedure

Recruitment and interviews were conducted in line with the Code of Ethics and approved by NHS ethics (REC ID: 20/LO/0082). All procedures were performed in accordance with relevant guidelines. Sampling was purposive, with an advertisement (Appendix 1) shared via social media, and a third sector support organization for parents. Couples were invited to take part in an interview focusing on their recovery from traumatic childbirth, and could contact the researcher to arrange a time and date for the interview. Interviews were conducted by the first author, a trainee clinical psychologist, via Microsoft Teams, and ranged between 1.5 and 2 h in length. All interviews were audio-recorded and transcribed verbatim; participants were provided with the opportunity to take a break or terminate the interview if needed. All couples were provided with a £10 voucher as a thank you for their support.

### Interviews

Participants took part in a semi-structured interview which was developed in consultation with a service user advisor and a specialist perinatal clinical psychologist (Appendix 2) with questions around the psychological symptoms of trauma after birth, and what helped or hindered recovery from these symptoms as a couple. The interview aimed to develop a narrative of the participants’ worldview of their situation, rather than a generalizable account of how other people may experience childbirth, seeking depth rather than breadth of understanding [17]. Informed by IPA, interviews contained minimal probing, and followed lines of inquiry relevant to what participants deemed important to discuss.

### Data Analysis

IPA used to analyse the transcripts. IPA was deemed appropriate given the aim of understanding the participants interpretation of their experience of traumatic childbirth. IPA stems from a phenomenological stance of double hermeneutics, giving credence to the researchers interpretation of the participants understanding of their experience. Interpretation and meaning is garnered from participants discourse, with the theoretical underpinning of IPA relating to the meaning participants make from their world experiences [18]. To preserve participant confidentiality all participants were assigned pseudonyms. The first author read each interview transcript multiple times, recognizing emerging themes and patterns, followed by re-reading the transcript and naming overarching categories that captured these themes. Further repetition of this process was required to select meaningful quotes and phrases substantiating the identified themes. This process was repeated for each interview transcript individually before pulling together identified themes across interviews. Coding was initially guided by the research questions, in line with Smith et al.’s [17] staged process, with the author identifying descriptive and linguistic comments. Further reading of transcripts led to the development of conceptual codes, which were reviewed and edited by the two remaining authors. The researcher’s own views and assumptions were regularly reviewed and discussed between authors as an integral part of the analysis process. This analysis and interpretations were supported by drawing on trauma theories of shattered assumptions [19, 20], emotional processing [21] and post-traumatic growth [22, 23]. The COREQ framework on qualitative reporting [24] was followed during write up of analysis and results.

### Reflexivity

The author undertaking interviews also experienced pregnancy and childbirth during the same period as the participants, her prior experiences and understandings of personal childbirth were discussed with the research team. This exercise was completed and revisited to challenge the first author’s pre-understandings and ensure pre-understandings did not dominate the discourse in interview or mislead the analysis of the participants experiences. The researcher did not personally experience childbirth as traumatic, nor experience any difficulties with mental health difficulties following childbirth, therefore was mindful to hold in mind and reflect on their own childbirth experience regularly when analyzing the data.

## Results

### Participants

Participants (described in Table 2) comprised of six heterosexual couples, aged between 20 and 40 years, in which at least one member of each couple reported experiencing the birth as traumatic. All participants were first time parents with a child under the age of 2 years. One couple had birthed twins, and all remaining couples had singular childbirths. All but one of the couples gave birth in the United Kingdom under the care of the National Health Service (NHS), the remaining couple gave birth under the French National Healthcare System.

**Table 2:** An introduction to participants

Alice & Ed	Alice and Ed are a White British married couple in their late 20’s, who have one 7-month old child,. Their trauma related largely to how health care professionals responded to them during the labour. Alice had a background in a healthcare profession
Susan & Peter	Susan and Peter are a White British married couple in their 30’s who have 10 month old twins. They described the birth as traumatic with intensive medical intervention, but the lasting trauma impacts come heavily from the poor aftercare they received. Susan has had 6 sessions of therapy and Peter is considering taking up individual therapy too. Susan returned to work when the twins were 6 months old.
Lucy & Jack	Lucy and Jack are a White British married couple in their late 20’s. This birth was their first child. Lucy’s birth involved an emergency C-section. Their son is 14 months old. They described a traumatic childbirth; privately Lucy reported receiving good after care.
Tash & Colin	Tash and Colin are a White British, married couple in their late 20’s and early 30’s. They have 1 10-month old child. Tash’s birth involved an epidural and a spontaneous vaginal delivery. Tash experienced health difficulties in her pregnancy, and consequently had a lot of healthcare appointments. She then experienced physical complications during labor, and postnatally was diagnosed with a a serious previously-undetected health condition with ongoing health consequences
Ivy & Stephen	Ivy and Stephen are a White British, married couple in their early 30’s. The birth they discussed was their first experience of childbirth, and their baby is now 10 months old. Ivy experienced instrumental intervention during her birth.
Polly & Adam	Polly and Adam are a couple in their 30’s with one son who is 2 years old. Adam is White French and Polly is White British. They live in France. Polly had an emergency C-section. She describes struggling with anxiety in the past. Polly reports having received brilliant health care and postnatal support in France.

### Findings

Four themes, each formed of subthemes, described how couples made sense of their birthing experience, and their process of recovery (Table 3). The focus lies on the emerging narrative surrounding the couples’ relationships to each other and their health care system. The four themes are described below.

Throughout analysis it became evident that mothers spoke more frequently than their birthing partners; as such exemplar quotes are predominantly from mothers. However, mothers often used ‘we’ pronouns to describe the experience, and the presence of the father during the interview will undoubtedly have affected their description of the experience, a concept which is discussed further later in the paper.

**Table 3:** Themes and Subthemes

Theme	1. We need validation	2. Feeling paper thin	3. This is a system failure	4. Birth trauma is always going to be a part of you
**Subthemes**	My voice was not heard	Disconnect from my emotions	Lost trust in the system	Advocacy and representation
	Responses from others compounded trauma	A need for Mum to be cared for	Retraumatizing experiences	My baby is OK!
	Am I going mad?	Shared experience versus sharing trauma	You’re gonna say I’m an unfit Mum	Growing my family
	Acknowledgement of system failure		Knowledge, control and guilt	Rediscovering our roles and identities

#### Theme 1: ‘We need validation’

All participants reported a need for validation of their physical and emotional pain from friends, family and healthcare professionals. Support received both during and after the birthing process sometimes contributed to couple’s distress. This emerged as most mothers feeling that their voice was not hear﻿d during their birthing experience. As Alice explains,

*‘I just wasn’t respected, there was no dignity. There was. I didn’t matter at all. I was done to, not done with.’* (Alice).

Alice’s experience resulted in her feeling that she was treated as an object rather than a human being. Respect and dignity were lacking in her treatment by healthcare professionals.

Fathers rarely spoke about their voice during the birthing process which could be interpreted as it being the mother’s story to tell.

In contrast, Polly spoke of the options she was given during in her post-natal care, which for her, promoted a sense of being heard by her healthcare team,

‘*it was all getting too much and then one of those… it was a student nurse and she just saw that I was upset. I was, and she just said, you know do you, would you want to be in touch with psychology team and I said yes and that was it*’.

For Polly, her distress was recognized and validated during the experience, whereas others, like Alice, felt this was overlooked.

For some participants, responses from others compounded their traumatic experienc﻿e, contributing to their distress. As Susan explains,

‘*When I did try speaking to them, I was crying and they just looked at me. They, they never said: “Are you OK? Why are you crying?”… instead they openly, you know, sniffed at me or and walked away, stormed off’.*

This demonstrates Susan’s initial difficulty in raising her need for support, and the lack of emotional response received by professionals around her, which could be seen as being treated as inhumane or disrespectful care by care providers. Many of the mothers attempted to seek support from their friends and family, whose comments were viewed as well-meaning, yet often invalidated their experience. As Alice explains,

*People try and make you feel better instead of validating you often, don’t they? And I need validation. That’s what I’m after, or I need someone just to listen and I don’t need you to fix it. We just need you to hear me and see me and, and... and hear my pain.*

Alice expresses a need for validation, which she represents as her own experience (‘*I need someone’, ‘I need validation’, ‘My pain’*), whilst also highlighting her partner’s need for support (“*we just need you*”). The distinction drawn between being heard and attempting to be ‘fixed’ demonstrates the simplicity in the support Alice feels she requires. Participants reported that close friends and family would attempt to ‘*fix things’*, as in Susan’s case,

*whilst they [the family] were sympathetic they didn’t understand - the common thing that we heard was ‘oh well, but you’re out now, you’re at home now. You’ve done. Your babies are fine. Just let it go, forget about it’ - and so we tried. I think that’s partly what drove the the burying it for for so long, because people were just telling us that, you know the medical professionals were telling us there was nothing wrong, and people that we knew socially were telling us, well, yeah, that’s a horrible experience, but time will heal everything.*

Susan’s narrative shows friends, family and healthcare professionals dismissing their concerns and consequently re-writing her and her partners experience, implying that the outcome of healthy babies should be enough to overcome the trauma felt. This rewriting of traumatic experience shows the lack of validation experienced when it was most needed.

Birthing partners, in this case fathers, were of paramount importance for all mothers in the recovery process by providing mothers with validation about their experience, as in Ed’s comment.

‘*You thought you maybe weren’t tough enough, which is ridiculous. ‘cause you’re tough as nails’* (Ed).

Mothers also validated their partners’ involvement in the process, as demonstrated in this exchange between Ivy and Stephen,

‘*I’m just trying to be… a bit of a rock’* (Stephen). ‘*It’s more than a rock. You’ve been like a mountain, even like a boulder… you’ve been. You’ve been incredible throughout this entire thing’* (Ivy).

Through these interactions, the provision of validation between the couple contrasted to the lack of validation experienced from outsiders to the relationship. However, whether these interactions and validation would occur organically, or were voiced due to the nature of the interview was unclear.

For mothers and their partners, a narrative emerged around feeling that they had failed their newborn through the traumatic childbirth, and the lack of validation and support from the health system appeared to reinforce this belief. As Ed described,

*It’s* [people’s responses] *kind of affected how you see yourself as a parent in that you feel like you’re not good enough as a parent*.

Ed’s comments show that the absence of validating comments not only reinforced the trauma experience for couples but also impacted on their sense of self with regards to parenting.

In direct contrast to Ed’s experience, Polly spoke of receiving support from her healthcare team in a timely manner,

*I rang the number and it actually - I remember kind of dreading having to make the phone call. But the people on the other end of the line were just really sweet and really nice, and just made it happen really quickly.*

Whilst like Susan, Polly speaks of the challenges of asking for support post birth, here, Polly’s healthcare team provided a timely response to her request for help. Simply, needing to ask for help made some participants feel like ‘not good enough’ parents.

For some couples, this lack of support, validation and distress culminated in experiences,

which we have drawn together in the subtheme ‘Am I going mad?’ For Susan, feeling invalidated left her questioning whether she was at fault for her emotional responses:

*Maybe I’m blowing this up. I said, I said it to her [*therapist*] the other day, I said I still feel really guilty, like I’m wasting your time, like there’s not really anything wrong with me?*

The lack of support from others left Susan feeling isolated and alone with her feelings, in turn causing her to feel guilt when accessing support. However, through support, and eventual validation from her therapist, Susan appears to reconcile her internalized blame;

*So yeah, for me, that it was simply someone saying, no, you’re.. you’re not OK. It’s fine not to be OK and there’s some you know and and and you’re not not lying basically.*

This demonstrates a time where Susan did receive the validation she, and other participants were seeking and the positive impact it had on her. This impact relieved the internalized self-stigma Susan had created, assuming others accusing her of lying.

Participants spoke of the experience of relief and validation when a system failure had been acknowledged either by the health care service or someone externally, which is the point when they were finally ‘heard’. Alice described that moment:

*We’re talking about a system failure. We’re not talking about you as an individual, or you know you having unrealistic expectations or you not behaving and doing what the system you know. This is a system failure. This is…It’s not on you.*

The acknowledgment of an external failure brought validation, and reduced blame on the parents. Alice’s powerful statement ‘it’s not on you’, demonstrates the blame she was holding for herself prior to the acknowledgement of a system failure. Likewise, Lucy’s experience of a private birth debrief held similar relief;

*‘it made me feel like I wasn’t mad and that what happened wasn’t very good and people who are in charge made mistakes, and it wasn’t my fault’.*

The idea of being ‘mad’ was raised again by Lucy, suggesting that participants sense of self was being altered by the invalidating experiences occurring, and only when the system was interrogated and held to account, could these couples reduce the burden of the self-stigma they were experiencing.

#### Theme 2: feeling ‘paper thin’

This theme explores how participants felt after their traumatic birthing experience. A common narrative was that the couples were left feeling vulnerable, and more susceptible to stress than before. The combination of having a newborn baby, and experiencing a traumatic birth, often left couples feeling unable to cope. One key aspect of managing these vulnerabilities was a need to ‘disconnect from their emotions’. Peter and his partner avoided seeking support for a long time, attributing their difficulties to the challenges of supporting a newborn. As Peter explained.

‘*We were exhausted and for a long while, although we were aware of things like the night terrors and the crying and bits and pieces we, we didn’t get to a point where we could begin to consider that actually something had been wrong for a long, a long while’.*

Peter demonstrates the avoidance of acknowledging the impact that birth trauma was having on them as a couple, despite being faced with the outward, more tangible psychological symptoms of trauma. Similarly, Polly describes her physical and emotional avoidance from tasks following birth trauma

*It’s hard to describe it, but just I couldn’t even look at the [baby’s cot] manual and even attempt it. It. My anxiety was so high that just even a basic task was just not even doable.*

Polly’s avoidance extended to the physical tasks involved in child raising, as her distress took a more physical form when finding herself unable to approach tasks due to anxiety. Couples frequently described noticing this period of vulnerability retrospectively in discussion together, rather than knowing this was happening at the time. It appears that couples experienced a period of emotional numbing in the early stages which acted as a protective factor whilst needing to adjust to life with a newborn. Partners highlighted their emotional numbing in their differing coping styles;

‘*for me I probably just kind of… put that in a box and like you say I was kind of in survival mode of just need to do this, need to do that. we get that done*. *You know you’ve had that real need to go back over things and try and re-process things.’* (Ed).

Ed’s description of putting the trauma in a box is a clear analogy outlining the difficulty that they faced when needing to raise a baby following birth trauma. The baby’s needs are inferred as the immediate task to address, and processing the traumatic experience was put on hold, seemingly unintentionally. Other couples experienced similar patterns, with partner’s being more likely to avoid the emotional connection to the trauma,

‘*I’m… stoic I I’m. I’m good at the emotional detachment from the event itself*’ (Peter),

Peter’s comment interestingly places positive connotation with emotional detachment, praising it as a useful defense mechanism in the process, and associating it with positive characteristics such as stoicism. Partners highlighted different ways of processing the event, involving engaging in sports and hobbies rather than talking about it,

‘*I’m not a talker…like I like to go to the gym and do judo. So that’s sort of a stress relief for me, and seeing friends there*’ (Colin),

‘*I think the thing I do I really obsessively were those blasted forms for filling in how much of the babies are eating and drinking’* (Peter).

These comments both demonstrate the partners’ tendencies to focus on practical tasks rather than speaking to the emotional experience itself, however whilst Colin frames this as a ‘stress relief’, Peter highlights his focus as more of an avoidance technique, demonstrating differing intention behind the action. Partners also raised their need increase their domestic role whilst mothers recovered emotionally from birth trauma, including childcare, as well as caring for their partner and general running of the home. Colin explains this as a potential catalyst for avoiding engaging emotionally with what was happening,

‘*I knew you was struggling, you know, physically but also I think I avoided picking up the psychological stuff just because of I had to do majority of the other things you know myself.*’ (Colin).

Here, Colin shows his inability to engage with emotions due to the overwhelming nature of chores he took on whilst his wife was struggling psychologically. Colin initially acknowledges that his wife was struggling, but denies his own struggles, showing a priority for his wife’s psychological wellbeing rather than his own.

For Stephen, the difficulty came from being required to have sole responsibility for their newborn before he felt ready, owing to Ivy being in hospital.

‘*there was a night or two where I was just really upset and I was really struggling because I thought I’d have a bit… a few weeks to kind of get up to speed and you know, change my share of nappies kind of supervised.*’ (Stephen).

Stephen here, talks about the role of his wife as his ‘supervisor’ in child raising, perhaps showing his uncertainty in his new role, and the emotional difficulties stemming from the inability to have support whilst ‘getting up to speed’. The lack of thinking space afforded to partners during this process may have contributed to the difference in gendered coping styles described. Partners also held a role in supporting mothers to re-connect with their emotions during this period; Stephen describes a difference in how Ivy relates to him post-birth.

‘*I think when you’ve had these issues before and you were letting it out on me and, having, having rage and stuff. It was kind of fine. Or you know, you know I could kind of cope with it, but you didn’t wanna be doing that.*’ (Stephen).

This observation by Stephen shows the beginning of growth and positive change following trauma, as he describes Ivy’s improvement in relating to him. Ivy in response, acknowledged the change as a result of appreciating the vast support that Stephen had provided her, and not wanting to display her previous coping style in front of their newborn,

‘*I didn’t want to be doing that. I didn’t want to do it to [baby] and I didn’t want to be getting upset at you ….I’m so hugely thankful for like the love and support that you have just kind of given me unconditionally throughout all of this*’ (Ivy).

Whilst showing a difference in their relational style, Ivy is also providing further validation to Stephen and using this as an opportunity to show gratitude to Stepehen for how he has supported her.

A consequence of emotional numbing, and in a context where families were struggling but not receiving support, a dialogue opened across participants that there was ‘A need for Mum to be cared for.’ Almost all mothers felt there was a lack of support, and that healthcare support that was received was aimed at the newborn child. Ivy spoke of the lack of support her and her partner received post birth:

*We had nothing in that first year. Nobody checked in on how I was doing. None of the midwives asked how I was after the birth*.

Ivy’s comment describes a sense of abandonment by health care professionals, indicating that she felt staff held a responsibility to check on her following birth trauma. Furthermore, Ivy indicates a sense of distrust in the midwives who did not enquire about her wellbeing despite witnessing what she went through; this further adds to a lack of validation as even the professionals who were present during the experience did not demonstrate care and compassion Ivy desired.

Mothers wanted to know about where they could get support and care they felt they really needed and deserved post birth trauma. Where postnatal care for the mother was felt inadequate, mothers often sought support from online peer groups, where the subtheme of ‘Shared experience versus sharing trauma’ became apparent. This is used to describe how couples often noticed that their vulnerability post-birth meant that they found it difficult to listen to other couples’ experiences of trauma. For example, Ed noticed that Alice did not always benefit from the group discussions with other families,

‘*Ed: It’s interesting ‘cause I hope you don’t mind me saying, you found those groups a bit 50/50. Some of them are really helpful and supportive and some of them really didn’t help.*

*Alice: Yeah.*

*Ed: Some of them really just made you like focus on the bad bits and like you know in not helpful way.*

*Alice: and I, I guess that’s the playoff of not accessing individualized support in that it’s not tailored to you and what’s helpful for somebody else, or what somebody else needs might not be what you need. Uh, and might not be helpful for you.’*

Ed was tentative about sharing something that Alice did not find helpful, however Alice agreed with his observation, expanding it by demonstrating the difficulty with accessing support that was not “tailored”.

Lucy and Jack found that by sharing the experience of birth trauma between them, they were able to make positive interpersonal changes in their relationship,

‘*We’re actually, I think, much better at communicating that beforehand. I would probably just, just bottled it up or not said anything or just carried on regardless, but I think we’re much better now at openly saying look this, this isn’t working or or whatever*’ (Lucy).

Lucy’s comment shows further positive growth that can emerge from experiencing a shared trauma between a couple, and that despite being through birth trauma, they are now able to address difficult emotions more openly with one another rather than avoiding the conversation. Lucy also noticed a shift in Jack’s responses to her difficulties,

‘*Jack knows the things that could potentially trigger me and he, he’s much better now than he ever was before we had children at recognizing when I need to have a break*’.

This shows interpersonal change in both directions within the relationship, and perhaps the impact that trauma has on supporting a couple to notice and respond to each other’s emotional responses more acutely.

Within Alice and Ed’s relationship there was a narrative that they had always had a good method of communicating with one another, which was protective when recovering from birth trauma and enabled them to work well together, however Ed highlighted the importance of being responsive to each other’s needs at a particular point in time,

‘*One of the things that’s been helpful is talking it through with each other. But I’ll put provision on that, that it’s finding the space to do it when you’re both able to do it, I think because of the sleep deprivation, because of the way that you handle it differently. Sometimes one of you needs to talk about it and the other one of you needs to not talk about it, right?*’ (Ed).

Here, Ed extends the process of being more attuned to his partner’s emotional responses to also acknowledging when his partner is not able to talk about the difficulties they are experiencing and allowing each other space within the relationship dynamic. Ed acknowledges differences in coping strategies and shows an ability of mentalization for Alice.

For some, social and professional support provided participants a relief from discussing trauma response within their couple; participants discussed decision making about the difference between personal and professional support and being a burden to loved ones. Ivy felt that the support she was seeking from her husband post-birth was becoming detrimental on their relationship,

‘*I knew that I I wanted to talk to someone else that wasn’t just Stephen I didn’t want to I I felt like… If I continue to burden Stephen with this it was going to have really detrimental effect on our relationship, like I’m his wife and his best friend and mother of his child and stuff like that like there there were… I didn’t want to just be the person he needed to look after, the person that had issues’.*

Here, Ivy describes the roles that she holds within her relationship with Stephen, and in particular the roles that Ivy holds as more important, or indeed more preferable and comfortable for her. Ivy felt that seeking support from Stephen resulted in her role within the relationship shifting, and described this as ‘detrimental’ showing a preconception for Ivy of what it means about her to require emotional support from a partner.

For other couples, seeking external help was more helpful in terms of the approach that a professional could take versus a loved one; Alice and Ed for example, debated that validating support needed to come from a professional source, Ed said.

‘*You need to hear it from someone else than me. Those sorts of things, and like the feeling of not being good enough, you need someone else to tell you ‘cause you think I just say it, ‘cause I’m your husband’.*

In this comment, Ed taps into the idea that a partner, by definition, should be invariably supportive, which could remove the authenticity of validation received by Alice from him. He uses this as evidence that a professional needs to be involved when rebuilding Alice’s sense of self and confidence as a parent post-birth trauma. For Polly and Adam, professional support was required, with the justification being that supporting one another was difficult due to the need to simultaneously look after their child, as Adam described,

‘*I don’t think I could be the best help. It would be very difficult for her to talk to me about, you know what’s going on in her head because I think we were focusing on at the same time on the baby*’.

The focus between the couple here lies on the baby, and feels as though the priority in the post-natal period between a couple needs to be on the child, and therefore focus on each other’s wellbeing is neglected somewhat.

#### Theme 3: this is a system failure

The theme arising with the most data representation was that of a poor relationship with the health system, culminating in a sense of having **‘Lost trust in the system’** post-birth. Participants generally reported plummeting standards in postnatal care, as summarized by Tash:

‘*the aftercare at the moment in our hospitals. We found it a bit…it was hard to stomach it, right?’.*

Tash singles out her aftercare as problematic, using the analogy of it being hard to digest. In particular, many participants spoke of the challenges of seeking support from a healthcare system that had let them down. As Alice, a health worker stated,

*‘I questioned whether I could even work for this system that hurts people in this way and therefore why would I want to go and seek support from the very system. I felt like the abused child asking the abusive parent for the love and care that they needed and I couldn’t bring myself to do that. I was like I’ll just go private.’.*

Alice’s complete lack of trust in a service that she worked for, shows the detrimental impact that her postnatal care had on her perspective of healthcare systems. The comparison of the health care system to an abusive parent draws a powerful image holding the healthcare professionals to account for the abuse experienced, implying a level of intent as with an abusive parent-child relationship.

After the traumatic birthing experience, and perceived lack of postnatal care, most participants has lost trust within the healthcare service, contributing to a delay in help-seeking. Ivy recounted,

‘*there was just nothing, there was a big lack of it. And I think that’s why it’s taking me this long to try and start … feeling more comfortable talking about obviously, and I’m getting the help that I certainly need’*.

Ivy’s comment shows her discomfort in disclosing how she feels, relating this to the lack of care provided, this is perceived as the lack of care and validation provided by health care services causing a discomfort and distrust in sharing her experience with others. Lucy described the poor care she received as more purposeful

I had some horrendous midwives who assaulted me, who were very rude to me and did things me that should never be done to anybody..

The description of Lucy, and other participants’ experiences is one of disrespectful and abusive behaviour by midwives, however Lucy was the only participant to name the experience as assault. The obstetric violence experienced by participants is rarely named as such despite the clear descriptions of disrespectful and abusive interactions. Participants also described services putting them through retraumatizing experiences, particularly the birth debrief sessions offered by maternity staff. Particular reference was made to the lack of thought given to the location,

‘*And then when we went for the debrief at the hospital nobody thought about the fact they stuck us in the room opposite the room I’d labored in. Uh, so I was a mess’* (Alice),

and the skills of staff holding these sessions, ‘*I know how dangerous like can be to be reliving trauma with someone who’s not skilled enough to support you through that*’ (Ed).

Alice recalling being a ‘mess’ gives a sense of how unclear and disjointed she felt after being made to revisit the location of her birth trauma, whilst Ed’s use of the word ‘dangerous’ shows how serious, delicate and important the resolution of birth trauma experiences were for him. Couples recounted high expectations for the debrief, but feeling dissatisfied afterwards, as Peter describes,

‘*cause that’s all the lady did. She just read out timestamp notes and that was it. I’ve no idea what the point of it was.*’.

Peter’s observation highlights the lack of empathy provided the medical intervention, where his and his partners needs were not being met, and the ‘timestamp notes’ are an insult to the parent’s time and vulnerability in seeking support.

For some couples, there was a perception of the power held by a healthcare system and the threat felt as a new parent attempting to navigate this system, summarized as ‘You’re gonna say I’m an unfit Mum**’.** For Susan, this was particularly prevalent when she was attempting to alert healthcare professionals that she needed help,

‘*I was so anxious to say there’s something wrong, I need help. Please don’t take my babies away - ‘cause I’m thinking you’re gonna say I’m an unfit Mum’.*

Susan’s experience highlights the power imbalance within the system between the mother and health care professionals, where mother’s feel that the extent of their trauma means their babies may be removed from their care. The power held within the system was particularly evident in the language used by professionals. For example, when Lucy attempted to access support from a perinatal service the words used, such as ‘*assess*’ and ‘*qualify*’ highlight the judgement and inaccessibility of the system;

they assessed me on the phone and said, oh, you know we have to assess you to see if you actually need it? So they assessed me and then said, Oh yes, you know you do qualify for help.

Many couples referred to the midwife six-week checkup appointment, when asked about their mental health; couples’ referred to this as a ‘*tick box loaded question’* (Alice), or as not being able to answer it honestly due to the threat of how services may respond; ‘.

*I’m not going to tell you about [*it*] because you’re going to send social services around - no I haven’t [*had suicidal thoughts*]. So I answered no. And and and so that that was it’* (Susan).

Susan’s lack of trust in the system led to her being unable to honestly answer a question about suicidal thoughts, as such, the question had no meaning or authenticity. The power imbalance is evident in the way she describes her fear of social services being sent around. On the contrary, couples described feeling less threatened and failed by services when they had access to information about support available; when parents held ‘Knowledge and control**’.** Lucy reflected,

*I wish I could have told myself that you know that there was help out there because actually when you look as a new parent like now, I have loads of places I can signpost people to, but I didn’t know any of those.*

Lucy’s comments here feel hopeful and positive following access to information; she describes having ‘loads’ of places for struggling parents to access, however the disappointment in not knowing these when she required them herself. For some couples, the lack of support related to a lack of understanding of what they were experiencing,

‘*I mean, I had all manner of weird things at that point, that we didn’t. We never thought it was trauma’* (Susan),

Susan’s description demonstrates the confusion felt during that time, using words like ‘weird’ to explain her experiences. For others, the difficulty related to not knowing where specifically to seek help for birth trauma,

‘*I’ve had therapy in the past for unrelated things, so I’m not, you know it’s not unfamiliar to me, but I still… This was completely different and, and I’d never felt like this, so I wasn’t sure where to go.’* (Lucy).

Here, Lucy shows that even as someone who has accessed therapy in the past, she was confused about what she was experiencing following traumatic childbirth.

#### Theme 4: ‘Birth trauma is always going to be a part of you’

All participants described the process of recovering from birth trauma as a couple, and understanding where they were on the path to recovery. Some couples used analogies, such as of recovery being like a book,

*‘it feels like you know if there’s a story it feels like half the pages have been torn out or chopped up and missing, and they’re all in the wrong order in and just feeling like, OK, well, we’ve put the book together as much as we can. And and here’s what we’re going to fill in the blanks with.’ (*Alice).

Alice’s analogy helps us to understand how confused and unclear the path to recovery is for her; she describes her story as feeling incomplete, being lost in a system of trying to understand her experiences.

Rather than determining recovery as an end point to achieve, frequent comments indicated that the experience is something that might always be with them,

‘*we discussed this the other night um, I don’t think you’ll ever get over fully over the trauma, because it’s always going to be part of you’* (Jack).

This powerful statement by Jack acknowledges the life altering experience that they have been through as a couple, demonstrating the sheer magnitude of birth trauma. Couples described channeling their trauma into productive activities, and a common theme included Advocacy and representation for others who might be in their position. Some participants, such as Peter, worried about people going through this experience in the future,

*cause our fear, our great fear was that, that thought that Susan had in the hospital will happen to somebody, somewhere, someday. And that is the reason for doing this stuff* [research]. *It’s because you just don’t want that to happen.*

Peter’s empathy towards his wife’s birth trauma extends to the general population, and his use of the words ‘great fear’ show the extent that he wishes for no one else to experience what she has experienced. The lack of control and power in his words is compensated by the regained power in taking part in research and advocating for people who could be in this position in the future.

A marker of recovery reflected by participants related to their baby’s progress and development, which placates participants’ concerns about the long-term implications of birth trauma. This is described in a subtheme named My baby is OK! As Ivy states,

*She* [baby] *doesn’t hate me and I’ve not broken her because of the awful stuff that.. that’s happened before.*

Ivy’s use of language shows the fear she held about the lasting impacts of her experience on her baby. The relief felt in her words, that her child has developed healthily is demonstrative of her perceived negative impact that birth trauma may have had on her child. Other participants felt that they were still on this journey of realization, but similarly described their babies’ wellbeing as a motivator for recovery, such as Susan,

‘*I need to get to a place where I reconcile myself that my babies are happy and that that’s fine. The job… we’ve done a fine job’.*

Susan’s description implies that she remains on the journey to reconciliation, but that her babies happiness is paramount to her recovery. Her description of their parenting as a ‘fine job’ seems to provide a modest account of raising happy twin babies.

A frequent topic during discussing recovery was the concept of Growing my family. For some couples this was the moment they identified that they were overcoming birth trauma, such as Lucy,

*when we kind of started discussing about having a second child, uh, I think when you’re starting to have those conversations, you’re definitely, you know, on on the path to to being healed*.

Another narrative that arose during discussion about living with birth trauma, was the couples’ desire to begin ‘rediscovering their role and identity**’** both individually and as a couple. For some, this related to a new identity, such as for Lucy,

‘*it* [birth] *completely changes you as a person uhm, you you know a baby is born and a mother is also born and and that saying is is so true’.*

Lucy’s powerful statement highlights the changes that she feels a person goes through when becoming a parent, however there is a sense of hope felt for the ‘new’ person that she is becoming. For others, this involved rediscovering parts of their pre-birth selves that they had lost touch with, such as Colin, who wanted to;

*‘get into a position where we can say you know, oh I wanna go out with a friend or something you you know and that’s fine. You can leave him with me, that’s fine’.*

Colin’s comments imply a yearning for normality that existed pre-birth and trauma, and that being the marker of recovery for him.

For others, the exploration into role and identity related to them as a couple, as described by Tash,

‘*we’ve sort of taken on our role as parents, but then we’ve kind of almost forgotten our role as a couple, and I think that’s that’s taken a huge back step as being husband and wife. We are Mum and Dad essentially, and I think we’ve sort of gotten a bit lost in that, and I think that’s what I’d like certainly achieve is being more of like the couple that we were before we had* [baby]’.

Similarly, Tash describes a preference to hold onto elements of their pre-parent relationship, and the importance of remembering their existence as a couple rather than solely parents. Other couples described parenthood as being an opportunity to co-construct a new role in unison;

‘*you are creating something new, and and you kind of. You can create that together and you can shape it. Yeah, and that can be a really positive empowering thing. And it’s not fixed. It’s something that’s developing*’ (Alice).

Alice shows the dynamic nature of discovery and recovery following birth trauma, discussing the developing roles they are co-constructing along the way. The description of creating something new holds hope and positivity for them as a couple.

## Discussion

This research brings a novel understanding of six couples’ experiences of recovering from traumatic childbirth within the past two years. Using an in-depth IPA approach the authors were able to make interpretations of the participants’ own sense-making of their lived experiences of jointly recovering from traumatic childbirth. This addresses a previous gap in knowledge into couples’ experiences of birth trauma, allowing consideration of ways clinical services can best support two-parent families during this time. Whilst the majority of comments made and analysed were spoken by mothers, the process of interviewing couples’ together and the presence of the partner has highlighted the couples’ systemic recovery from birth trauma. Many mothers used the term ‘we’ to describe what they experienced, and in some instances, spoke on behalf of their partner to describe their shared experience. Existing literature around birth trauma suggests that when partners are interviewed alone, they identify that their experience is not justified, or that it is not ‘their story to tell’ [25, 26] as such, our approach adds a novel perspective to the current birth trauma literature by understanding the systemic couples experience together.

Participants demonstrated experiences with strong narratives emerging around their relationship with the health care systems as well as the effect on interpersonal relationships. Participants demonstrated a lack of security in their position in relation to health care professionals, and a need for enhanced validation during a time when they felt most vulnerable. Many of the experiences that participants described were of obstetric violence from healthcare professionals; although the intent may differ from a typical abusive relationship, the described behaviour of healthcare professionals were nonetheless disrespectful and abusive. The experience of birth trauma left participants feeling less resilient than their ‘usual selves’, and the proceeding interactions with health care staff, friends and family therefore had a larger effect on them. This is concordant with established theories of trauma symptom development [27], whereby trauma stimuli such as interactions with staff continue to impact the couples’ negative appraisal of their experience thus resulting in dysfunctional cognitive strategies such as attempted avoidance and leading to continued reliving of the trauma experience. Power and threat positions between participants and health care systems were also described, with participants describing feeling out of control of their own experiences, leading them to question their emotional responses. Moving on from the traumatic experience centered around allowing some release of control, or being validated by external agencies that their experience was due to a system failure. For some, part of their recovery involved moving beyond their distrust in healthcare systems by disproving this assumption and accessing adequate support. However, some participants ultimately delayed access to support due to a breakdown in trust with services. This response is in line with the established shattered assumptions theory [19, 20] where the assumption generally held by participants before their birth trauma experience was one of trust in a system that would adequately support them.

The recovery of the couples can be interpreted through established trauma theories; in the current study participants identified an avoidance or inability to identify trauma difficulties during the first weeks to months of their newborn’s life. This is an inherent difficulty in the treatment of trauma following childbirth, as parents are required to ‘survive’ the initial days adjusting to life with a newborn, which participants’ described leads to an unintentional avoidance of some trauma stimuli such as avoiding connecting with their difficult emotions about the trauma, or avoiding tasks such as building baby furniture. This avoidance is described in the Emotional Processing Theory [21] which considers that emotional processing is key in the recovery and evasion of long-term pathology related to trauma. Impairments in emotional processing, such as avoidance, are proposed to lead to long-term trauma symptom development. Furthermore, parents in this study entered the childbirth process with differing knowledge of birth trauma and levels of trust in services, but a strong theme surrounded the cognitive shattering of the belief in the care and support that the health service will provide for them during a time of need. This is in line with the ‘shattered assumptions’ theory [19, 20] describing that humans hold consistent, core assumptions or internal representations about themselves and the world, which when challenged or ‘shattered’ can lead to the development of trauma symptoms. The current study identifies that this is relevant for new parents, particularly in relation to their relationship with healthcare services.

Participants described the fundamental changes to their personalities and actions following their birth trauma experience, in terms of the desire to advocate and represent other people who may follow their path. There was a narrative of increased compassion for people who may follow in their footsteps. This relates to the theory of post-traumatic growth coined by Tedeschi & Calhoun [22, 23] and widely described in trauma literature cross-culturally, describing positive change following a trauma incident. In the case of the current research, post-traumatic growth appears to be experienced both intrinsically through increased compassion for themselves and others, and better communication skills with their partner, as well as practically through the support for other people.

### Strengths and limitations

The current exploration of couples’ experiences of recovery from traumatic childbirth focuses on the couples’ accounts; a novel perspective within the evidence base to date. Due to the nature of IPA, there is an element of interpretation that has been made by the researcher.

Participants were not diagnosed as having experienced birth trauma or posttraumatic stress by a mental health clinician, which may vary the homogeneity of participants’ experiences. However, in each couple at least one member reported trauma symptoms which appeared to be consistent with the DSM criteria [1] for post-traumatic stress disorder.

### Clinical and research implications

An overarching implication is the evident development of trauma psychopathology being compounded by the language and communication used by health care professionals, which in this case of this study, often fell under the remit of disrespectful and abusive care by professionals. This was not just contained to the labor and birth itself; perceived abusive behaviour and language used post-birth had a significant impact on participants’ overall sense of their ability to be a ‘good enough’ parent in the context of a traumatic birth. Training on communication styles is essential for all health care professionals working with parents at all stages of pregnancy, childbirth and post-natal recovery including an understanding of emotional processing following trauma and validation techniques. This communication should aim to support parents to feel validated in their experience and response, and overcome the sense of guilt reported by couples following traumatic childbirth. Furthermore, to navigate the issue of trust in services, and encourage a timely and honest approach to support, services need to be commissioned with a trauma-informed approach in mind. Midwives are not routinely provided with trauma-informed care training [28] which could prevent compounding a re-experiencing of trauma for couples. This study demonstrates that trauma-informed care in maternity services could involve considering the loss of trust that parents may feel in relation to health care services, as well as issues pertaining to location of services, staff training and a sense of control for service users. A key point arising from this study is the element of control gained from knowledge provided about service options, and what interventions may entail. This reduces perceived threat from parents around consequences of disclosing their mental health difficulties postnatally and dissipates issues of client-service trust. Services further need to consider couples intrapersonal coping styles and skills, building on these within a therapy context to promote recovery and move towards post traumatic growth [22, 23]. This includes considering factors such as the couples’ need to speak to someone external for support in order to reduce the impact of trauma experienced on their interpersonal relationship as well as a consideration of practical factors such as who cares for the child whilst therapy is in progress.

## Conclusion

The current research explores couples’ worldview of their recovery from traumatic childbirth with a focus on what aids and hinders recovery within the systemic relationship. This study identifies the importance of language used by healthcare professionals, and the need to support parents to feel validated, particularly to reduce feelings of guilt resulting from their experience. A knowledge of services and control over intervention is important for mothers and their partners when considering recovery, and parents’ highlight the need for external support in order to reduce the burden on their interpersonal relationships. The current findings have clear implications for the development and adaptation of perinatal mental health services to provide trauma-informed support for parents in the post-natal period, with validation central to the approach.

## Electronic supplementary material

Below is the link to the electronic supplementary material.

Supplementary Material 1

## Data Availability

Analysed data is provided throughout the manuscript in tables and in the form of participant quotations. Full, anonymised interview transcripts are held by the University of Surrey in line with the Data Protection Act (2018).
