# Supplementary material for: An interpretative phenomenological analysis of the experience of couples’ recovery from the psychological symptoms of trauma following traumatic childbirth
Source: BMC Pregnancy Childbirth. 2022 Oct 29;22:798. doi: 10.1186/s12884-022-05091-2 (PMC9617293; doi:10.1186/s12884-022-05091-2)
Supplement: Supplementary file 1 — Supplementary Material 1 [file 12884_2022_5091_MOESM1_ESM.docx]

**An interpretative phenomenological analysis of the experience of couples’ recovery from the psychological symptoms of trauma following traumatic childbirth**

**Appendix 1; Research advert to recruit participants**


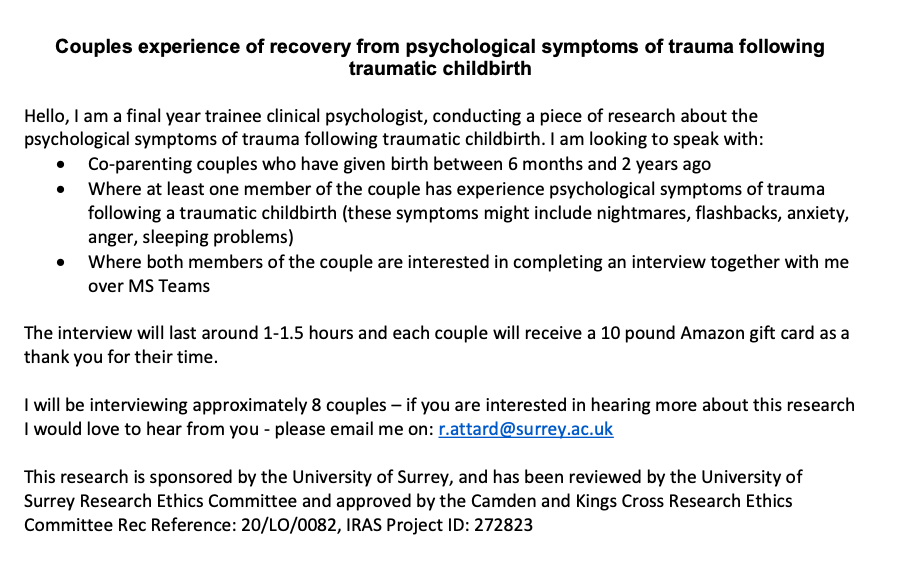


**Appendix 2; Interview Schedule**

-Introduce the topic with a brief recount of the information sheet, including:

Confidentiality

participants’ rights to withdraw/have a break at any time during the interview.

-Complete informed consent

-Not like therapy

*Opening statement*: Thank you again for agreeing to take part in this study.

I’m interested in hearing about both of your experiences following the birth of (name baby/child).

You have indicated that one or both of you found this experience traumatic in some way. During this interview I would like to find out more about how this has impacted on you since then.

In particular, I am interested in finding out more about your recovery from this experience – so being able to understand what has helped or perhaps not helped you towards being able to overcome this experience.

I understand that there may be similarities and differences in your experiences, and whilst you are unlikely to have the same views or emotions about the experience, I would be interested in hearing how each of you have perceived this. So, I’m hoping that I can hear from both of you during this interview, to understand your individual experiences, but also to think with you both about how you have found this as a couple.

Q1: We’re going to be focusing on your recovery from psychological symptoms/mental health distress resulting of trauma in this interview, however before we begin, it might be helpful to hear some background to your experience; please can you tell me a bit about your experience of childbirth?

Q2: When did you first notice the psychological symptoms of trauma/that you were finding it hard to recover emotionally from your childbirth?

Prompt: Who noticed these symptoms? (What was the other persons view on this?)

How long after childbirth did this occur?

How did these difficulties show themselves?

Did you notice any impact of these symptoms?

- {If COVID-19 is mentioned as a reason} – Do you think this would have felt different if you gave birth before the pandemic?

Q3: When did the decision occur to get support?

Prompt: Was there anything that influenced that decision to get support?

Was there anything that influenced what support you believed might be most helpful?

Whose idea was this? What did the other person think about that?

Was there a specific moment you can identify that you thought you ought to get support?

Did you have similar or different views on getting help?

Did you have similar or different views on what sort of support may be most / least helpful?

- {If COVID-19 is mentioned as a reason} – Did the pandemic have any impact on your experience of getting support?

Q4: When one/ both of you decided support was needed/ wanted what did you do with that initially?

Prompt: And then what happened?

From family and friends?

From NHS services?

From voluntary sector organizations?

From websites? From social media?

Q5: Thinking about it now, was there anything that you found particularly helpful?

Q6: And was there anything that you found unhelpful in your recovery?

Q7: Looking back, is there anything that you wish you’d known earlier in your recovery from childbirth, that you now know or that has really helped you to overcome your difficulties?

Prompt: Is there anything that you wish had been done differently?

Q8: What does recovery from these symptoms look like to you?

Prompt: As individuals? As a family?

Q8: Was there anything else that either as individuals or as parents that helped you recover?

Prompt: Is there anything that specifically helped you as a couple? As parents?

Q9: Was there anything else that you think did not help you in your recovery?

Prompt: And the other person?

Q10: Is there anything else important that you haven’t had a chance to say?
